# Characterization of Metabolites of Leonurine (SCM-198) in Rats after Oral Administration by Liquid Chromatography/Tandem Mass Spectrometry and NMR Spectrometry

**DOI:** 10.1155/2014/947946

**Published:** 2014-02-24

**Authors:** Qing Zhu, Jinlian Zhang, Ping Yang, Bo Tan, Xinhua Liu, Yuanting Zheng, Weimin Cai, Yizhun Zhu

**Affiliations:** ^1^School of Medicine, Nantong University, 19 Qixiu Road, Nantong 226001, China; ^2^Department of Pharmacology, School of Pharmacy, Fudan University, 826 Zhangheng Road, Pudong District, Shanghai 201203, China; ^3^Department of Clinical Pharmacy, School of Pharmacy, Fudan University, 826 Zhangheng Road, Pudong District, Shanghai 201203, China; ^4^Analytical and Testing Center, School of Pharmacy, Fudan University, 826 Zhangheng Road, Pudong District, Shanghai 201203, China

## Abstract

Leonurine, a major bioactive component from *Herba Leonuri*, shows therapeutic potential for cardiovascular disease and stroke prevention in some preclinical experiments. The aim of this study is to characterize metabolites of leonurine in rats using high performance liquid chromatography coupled with tandem mass spectrometry (HPLC/MS/MS). The chromatographic separation was performed on an Agilent ZORBAX SB-C18 column using a gradient elution with acetonitrile/ammonium acetate buffer (10 mM, pH 4.0) solvent system. An information dependent acquisition (IDA) method was developed for screening and identifying metabolites of leonurine under positive ion mode. Compared with control, the interesting compound in the extracted ion chromatogram (XIC) of the *in vivo* samples was chosen and further identified by analyzing their retention times, changes in observed mass (Δ*m/z*), and spectral patterns of product ion utilizing advanced software tool. For the first time, a total of three metabolites were identified, including two phase II metabolites generated by glucuronidation (M1) and sulfation (M2) and one phase I metabolite formed by O-demethylation (M3). Finally, the lead metabolite M1 was isolated from urine and its structure was characterized as leonurine-10-O-**β**-D-glucuronide by NMR spectroscopy (^1^H, ^13^C, HMBC, and HSQC).

## 1. Introduction

Leonurine (SCM-198), 4-guanidino-n-butyl syringate, is a major biologically active component isolated from extract of *Herba Leonuri* (Yi-Mu-Cao), a Chinese traditional herb since ancient times used for treatment of gynecological and obstetric ailments [1], which in the past thirty years was reported to be used in the treatment of myocardial ischemia [2, 3] and cerebral ischemia [4, 5]. Nowadays, leonurine is available in our laboratory by chemically synthesis method and is being studied in preclinical phase. Studies have shown that leonurine has versatile pharmacological effects including uterotonic action [6], antiplatelet aggregation [7], and relaxation of vascular contractile responses [8]. More recently, it has been reported from *in vitro* experiments that leonurine could attenuate apoptosis in H9c2 rat cardiac muscle cells induced, respectively, by hypoxia, doxorubicin, and hydrogen peroxide which predicted its potential cardioprotection ability [9–11]. Evidence from animal studies demonstrated that leonurine exhibits cardioprotective effects both on the acute and chronic myocardial ischemia model in rats and the mechanism may be related to its antioxidation, antiapoptotic effect, and cardiac fibrosis prevention [12–15]. Moreover, it has proved that leonurine has neuroprotective activity against ischemic stroke and cerebral ischemia/reperfusion model in rats through antioxidative effect and regulation of mitochondrial function [16–18]. These findings have shown that leonurine has therapeutic potential for cardiovascular disease and stroke prevention.

Generally, xenobiotics accessed into living creatures will be eliminated from the body by enzymatic biotransformation which contributes to the bioavailability, pharmacokinetics, and pharmacodynamics. Drug metabolism is also one of the significant factors underpinning the failure of new drugs in preclinical stage [19]. So, it is critical to know the metabolic profiles of leonurine during drug discovery and development. However, no publication involving biotransformation of leonurine *in vitro* and *in vivo* has been reported so far. Recently, high performance liquid chromatography coupled with ion trap mass spectrometry (HPLC/MS/MS) has usually become a powerful technique for the metabolite identification because of its high sensitivity and specificity [20, 21]. So, this work is focused on identification of metabolites of leonurine in rat biological samples such as plasma, urine, feces, and bile after oral administration using HPLC/MS/MS method for the first time. In the present study we aimed to characterize metabolite profiles of leonurine and to describe the possible metabolic pathways in rats.

## 2. Materials and Methods

### 2.1. Chemicals and Reagents

Leonurine was chemically synthesized as previously reported (purity > 98% as determined by HPLC) [22]. HPLC grade acetonitrile, methanol, ammonium acetate, and acetic acid were purchased from Fisher Scientific (Pittsburgh, PA, USA). Deionized water was obtained from a Milli-Q plus Ultrapure Water System (Millipore, Shanghai, China) and used for the preparations of all solutions. All other chemicals were at analytical grade and were provided by Sinopharm Chemical Reagent Co.Ltd. (Shanghai, China).

### 2.2. Instrumentation

The HPLC/MS/MS system used consisted of an Agilent 1200 series HPLC system including a quarternary pump, a vacuum degasser, an autosampler, and a column oven (Agilent Technologies, PaloAlto, CA, USA) and a 4000 Q-TRAP linear ion trap triple-quadrupole mass spectrometer equipped with a TURBO-Ionspray source (Applied Biosystems Sciex, Foster, CA). Data acquisition and processing was carried out by Analyst 1.5 and Lightsight 2.2 software (Applied Biosystems Sciex, Foster, CA). The semipreparative HPLC system was composed of a Waters 600 liquid chromatography apparatus equipped with an autosampler and a Vis/UV detector (Waters, Milford, MA). A Bruker Avance 400 NMR spectrometer (Fallanden, Switzerland) was used for structure authentication.

### 2.3. Animals and Drug Administration

Male Sprague-Dawley rats weighing 200–250 g were provided by Sippr-B&K laboratory animal Corp. Ltd. (Shanghai, China). Rats were housed in separate metabolic cages and fed standard laboratory food and water to adapt to the housing environment for 5 days before experiment. Prior to administration, rats were fasted over 12 h but with access to water. All studies on animals were in accordance with the guidelines of the Committee on the Care and Use of Laboratory Animals in China. Leonurine was evenly dispersed in 0.5% sodium carboxymethyl cellulose (CMC) solution (3 mg/mL) for oral administration.

### 2.4. *In Vivo* Samples Collection and Preparation

For plasma sampling, six rats were administered by gavage at a dose of 30 mg/kg. Blood samples from angular vein were collected into heparinized tube at 0.083, 0.167, 0.333, 0.5, 0.75, 1, 1.5, 2, and 4 h after oral dosing. Plasma samples were prepared by centrifugation of blood samples at 3000 ×g for 10 min. Plasma samples were pooled in a time proportional manner to obtain a 0 to 4 h representative pooled sample for each rat [23]. An aliquot of 0.3 mL of pooled plasma sample was mixed vigorously with 1 mL of acetonitrile-methanol (v/v, 2 : 1) and then centrifuged at 10,000 ×g for 10 min to remove protein. The supernatant was evaporated to dryness under nitrogen stream at 40°C. The residue was reconstituted in 0.3 mL of water-acetonitrile (90 : 10). Urine and feces samples were collected from 0 to 16 h after oral administration of leonurine to six rats (30 mg/kg). Each urinary sample (1 mL) was mixed with 1 mL of 50% methanol, vortexed thoroughly for 1 min, and then centrifuged at 10,000 ×g for 10 min to remove debris. Feces samples were homogenized in 80% methanol to generate 10% homogenates and the homogenates were further sonicated for 5 min and centrifuged at 10,000 ×g for 10 min. For bile sampling, six rats were fixed and anesthetized by intraperitoneal injection of 7% chloral hydrate (0.5 mL/kg). The common bile duct was cannulated with polyethylene tubing for collection of bile. Bile samples were collected from 0 to 8 h after oral dosing of leonurine (30 mg/kg). The procedure of bile sample preparation was the same as plasma sample preparation. Blank plasma, urine, feces, and bile samples were collected before administration and were served as blank controls. The biological matrixes spiked with leonurine were also used as controls to exclude the false metabolites from impurities in drug. All the control samples were pretreated parallel to the *in vivo* samples. For all the prepared samples, an aliquot of 5 *μ*L was injected into the HPLC/MS/MS system for metabolite screening.

### 2.5. HPLC/MS/MS for Screening Metabolites

The chromatographic separation was performed on *a* ZORBAX SB-C18 column (150 mm × 2.1 mm, 5 *μ*m, Agilent, Palo Alto, CA) maintained at 27°C at a flow rate of 0.25 mL/min. Mobile phase consisted of ammonium acetate buffer (10 mM, pH 4.0) (solvent “A”) and acetonitrile (solvent “B”). The analytes were eluted using the following gradient program: initial composition was 10% B followed by a linear gradient from 10 to 50% B within 6 min, then 50% B for 3 min, a rapid increase from 50 to 90% B in 6 sec, 90% B for 2 min, and then back to 10% B followed by 7 min equilibrium before the injection of next sample.

Mass spectrometric data were obtained by using information dependent acquisition (IDA) experiments under positive ion mode. In IDA experiment, multiple reaction monitoring (MRM) scan was used as a survey scan and if signals in the survey scan exceeded 200 counts per second (cps), the enhanced product ion (EPI) dependent scan was triggered. The EPI scan rate was 4000 amu/sec and a scan range of 100–1000 amu was selected. The mass tolerance was set to 250 mmu and resolution of Q1 was set to unit. Dynamic exclusion criteria were set for 2 sec after 3 occurrences. The collision energy (CE) was set at 35 eV with a CE spread of 15 eV. The declustering potential (DP) was set at 90 V, and dynamic fill time (DFT) function was used. Other parameters were set as follows: curtain gas, 20; ionspray voltage, 5500 V; temperature, 400°C; Gas 1, 45 psi; Gas 2, 50 psi; CAD gas, high.

### 2.6. Leading Metabolite (M1) Preparation and NMR Spectroscopy

A large quantity of major metabolite was needed to confirm the structure by NMR analysis. Eight healthy male rats were administrated by gavage of leonurine at a dose of 100 mg/kg, once per day for 7 days, and the urine samples in these days were fully collected and stored at −80°C. Urine samples were lyophilized and then redissolved in 80% methanol. After filtration and centrifugation, the supernatant was subjected to the semipreparative HPLC system and performed on a Waters SunFire Prep C18 column (10 × 150 mm, 5 *μ*m, Waters, Milford, MA) at a flow rate of 4 mL/min. The detection wavelength was set at 265 nm. A MeOH-H_2_O solvent system was used as mobile phase and a gradient elution program was performed as follows: 0–2 min, 22% MeOH (v/v); 2–5 min, 22–40% MeOH; 5–8 min, 40–22% MeOH; 8–15 min, 22% MeOH. The eluent containing metabolite M1 at elution time range of 7.0–7.4 min was collected and then concentrated by vacuum distillation. The prepared metabolite M1 was purified and determined on a Waters SunFire C18 column (4.6 × 150 mm, 5 *μ*m, Waters, Milford, MA) using methanol-10 mM ammonium acetate buffer (pH 4.0) (35 : 65, v/v) as mobile phase at a flow rate of 1 mL/min. Finally, about 10 mg of M1 was obtained and further confirmed by ^1^H NMR (400 MHz), ^13^C NMR (125 MHz), HMBC (Heteronuclear Multiple Bond Coherence) and HSQC (Heteronuclear Single Quantum Coherence) spectra in D_2_O.

## 3. Results

### 3.1. Mass Spectra Analysis of Leonurine

**Figure 1:**
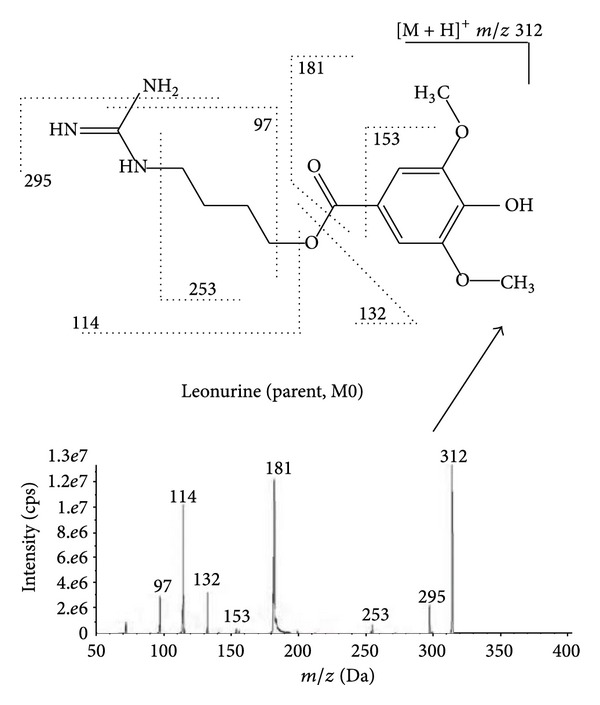
MS^2^ spectra and fragmentation pattern of leonurine (parent).

In order to better understand the MS^*n*^ spectra of the metabolites, it is necessary to examine the MS^*n*^ fragmentation pattern of parent drug. Due to the presence of guanidino group in the chemical structure of leonurine, the positive ion mode was selected. The protonated molecular ion showed a predominant ion at *m*/*z* 312 in the full scan mode. The base peak in the product ion spectrum was observed at *m*/*z* 181, associated with the cleavage of the ester bond Other major fragment ions at *m*/*z* 153, 132, 114, and 97 and fragment ions with very low abundance at *m*/*z* 295, 253 were also observed. The MS^3^ scan showed that the product ions at *m*/*z* 153 and 97 were generated, respectively, from the MS^2^ product ions at *m*/*z* 181 and 114. The MS^*n*^ spectra and the fragmentation pattern of leonurine were showed in Figure 1. In metabolites identification, if the compound showed these characteristic product ions or corresponding change in observed mass, it could be suggested as the metabolite of leonurine.

### 3.2. Metabolites Screening and Identification

Recently, a new linear ion trap-triple quadrupole mass spectrometer (Q-trap) was developed for screening and identifying metabolites which can provide not only traditional triple-quadrupole MS/MS scan including neutral loss (NL), precursor ion (PreI), and multiple reaction monitoring (MRM) scan but also linear ion trap MS scan such as enhanced MS (EMS) and enhanced product ion (EPI) scan. In addition, an EMS, NL, PreI, or MRM scan can serve as a survey scan to trigger IDA of EPI spectra. The IDA mode clearly increases the amount of information obtained from a single run [24]. MRM is an especially powerful technique for analyzing drug metabolic pathways which can detect the parent drug and multiple metabolites simultaneously with high sensitivity and specificity in biological samples. So, in this experiment, we utilized the Q-trap feature and used an IDA mode of MRM-EPI for screening and identifying metabolites of leonurine. Based on the MS^2^ spectra of leonurine, the two major product ions at *m*/*z* 181 and 114 were used to automatically generate theoretical mass transitions for MRM survey scan. Compared with the chromatograms of blank control and leonurine spiked matrix control, the interesting compounds in the extracted ion chromatograms (XIC) of the *in vivo* samples were screened out and further identified by comparing their retention times, changes in observed mass (Δ*m*/*z*), and MS^2^ spectra of product ion with those of parent drug utilizing Lightsight 2.2 software. Because no standards of metabolites were available for quantitation, peak area (counts) of each metabolite was recorded under the optimal MRM transition to calculate its percentage against total counts (including parent) by using area normalization method.

**Figure 2:**
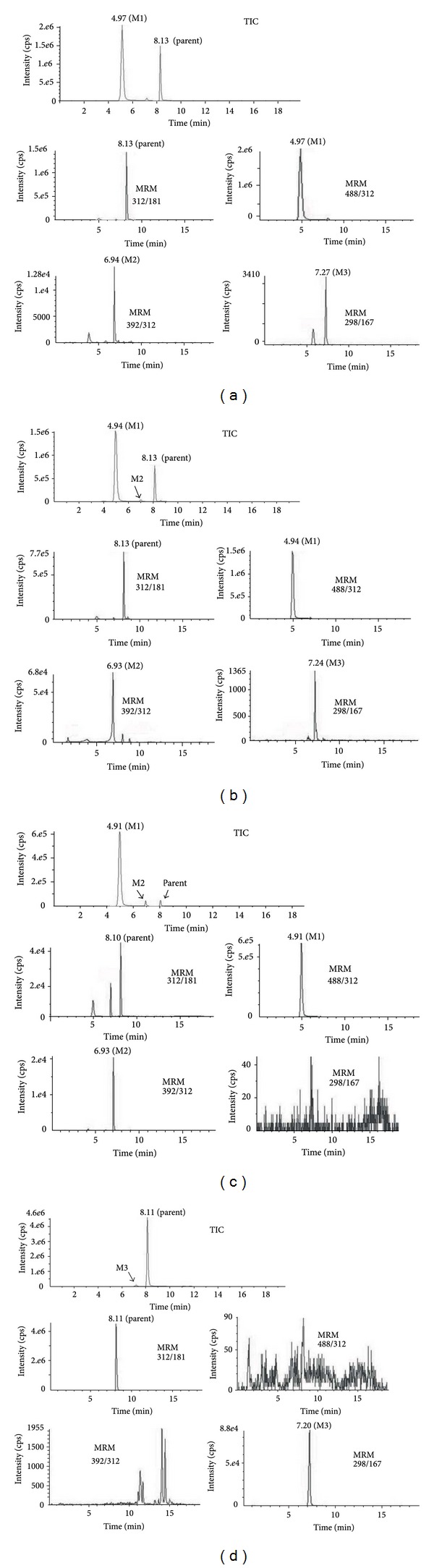
Representative extracted ion chromatograms for metabolites (M1–M3) in various biological samples after oral dosing of leonurine (30 mg/kg). TIC: total ion current chromatogram. (a) Bile samples; (b) urine samples; (c) plasma samples; (d) feces samples.

A total of three metabolites (M1–M3) of leonurine in rat were screened out and identified by using this method and the metabolic profiles in various rat biological samples were summarized in Table 1. We can observe all the three metabolites (M1–M3) in urine and bile samples, whereas two metabolites (M1, M2) were detected in rat plasma after oral administration. Only one metabolite (M3) in feces was observed in rat after oral dosing. The representative extracted ion chromatograms for the three metabolites in biological samples were showed in Figure 2. The mass spectra data and proposed structures of leonurine metabolites in rat were shown in Table 2.

The compound (MRM 312–181) eluted at the retention time of 8.1 min was identified as parent drug (M0) because its retention time, MS^*n*^ spectra of the protonated molecular ion at *m*/*z* 312 were identical to those of the leonurine standard.

**Figure 3:**
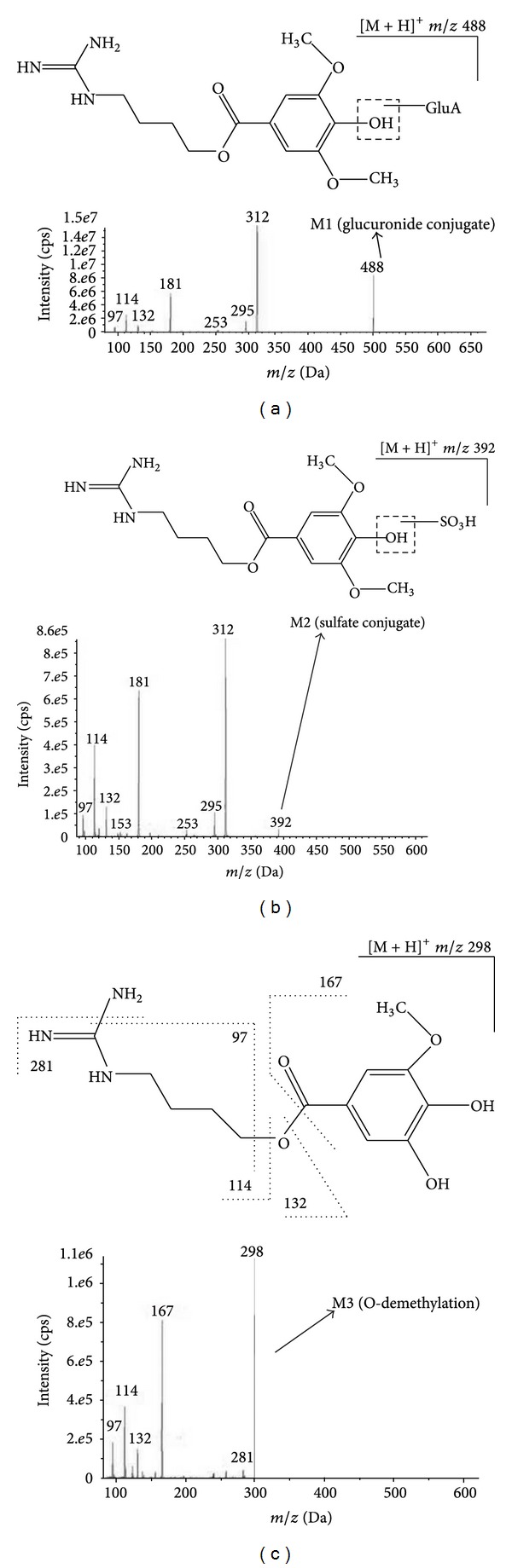
The MS^2^ spectra and fragmentation pattern of three metabolites. (a) M1; (b) M2; (c) M3.

Metabolite M1 (MRM 488–312) was eluted at the retention time of 4.9 min, showing protonated molecule ion at 488 Da, which is 176 Da more than that of parent. The MS^*n*^ spectrum showed their main product ion at *m*/*z* 312 (loss of the glucuronic acid moiety) and other fragment ions such as *m*/*z* 295, 253, 181, 132, 114, and 97 which were the same as those of parent (Figure 3(a)). These results indicated that M1 was glucuronide metabolite of leonurine.

Metabolite M2 (MRM 392–312) was observed at the retention time of 6.9 min, with protonated molecular ion at *m*/*z* 392 Da, 80 Da (equivalent to a sulfate unit) more than that of parent. Their main product ion was shown at *m*/*z* 312 corresponding to the loss of the sulfuric acid moiety and other product ions at *m*/*z* 295, 253, 181, 132, 114, and 97 were identical to that of parent (Figure 3(b)). From these results we can infer that M2 was sulfate conjugate of leonurine. In general, sulfate conjugation with phenolichydroxyl or alcoholichydroxyl was more conventional, so we tentatively assigned M2 as an O-sulfate conjugate, leonurine-10-O-sulfate.

Metabolite M3 (MRM 298–167), which was eluted at the retention time of 7.2 min, showed a protonated molecular ion at *m*/*z* 298, 14 Da less than that of parent drug equivalent to a loss of a methyl group. The MS/MS spectra of M3 showed product ions at *m*/*z* 281 (loss of an amino group) and 167 (formed by cleavage of ester bond) which were also 14 Da less than corresponding fragment ions present in parent at *m*/*z* 295 and 181, respectively. Furthermore, the characteristic fragment ions of parent at *m*/*z* 132, 114, and 97 were also observed in that of metabolite M3 (Figure 3(c)). These results indicated that M3 is generated by demethylation of one of the two methoxyl groups in parent. Thus, M3 was identified as an O-demethylated leonurine and its possible structure is 4-guanidinobutyl 3,4-dihydroxy-5-methoxy benzoate.

### 3.3. Lead Metabolite (M1) Preparation and Characterization

Among all the three metabolites, M1 was the absolutely predominant metabolite *in vivo*, with the percentage against total counts at 94.59% in pooled plasma, 77.90% in urine, and 80.38% in bile after oral dosing. Thus, it is quite necessary to figure out the authentic structure of M1 and further investigate its bioactivity.

**Figure 4:**
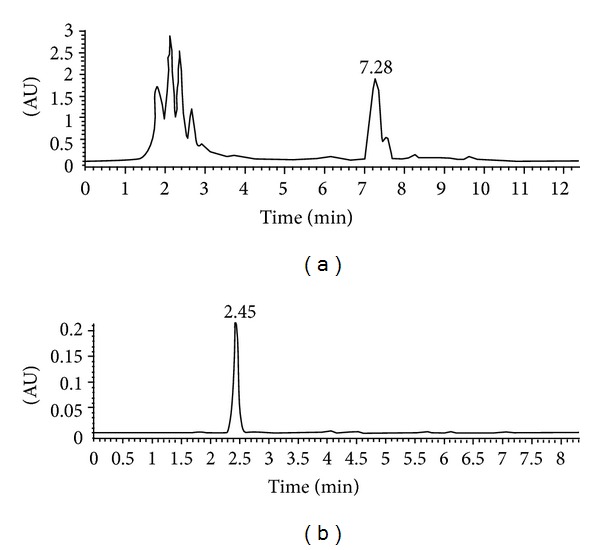
The semipreparative HPLC chromatogram of metabolite M1 in urine sample after intragastric administration of leonurine (a) and the purity analysis of the metabolite M1 acquired by semipreparative HPLC method (b).

**Figure 5:**
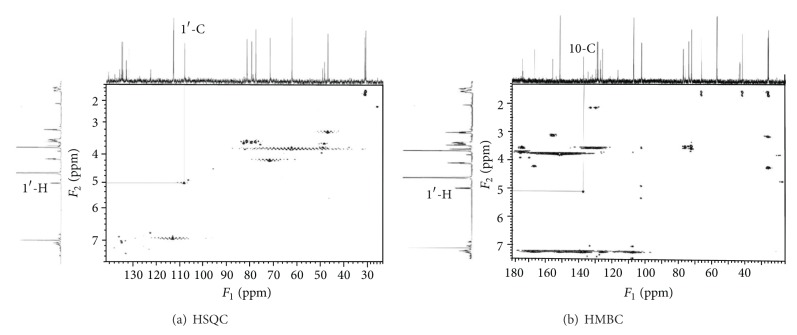
The HSQC (a) and HMBC (b) NMR spectra for metabolite M1.

**Figure 6:**
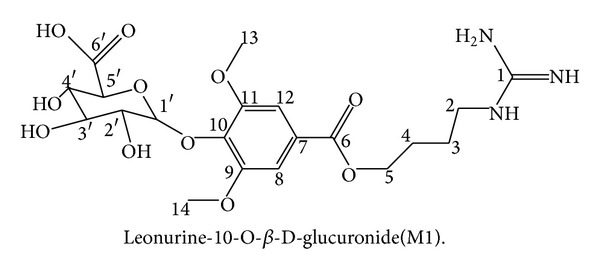
The authentic structure of metabolite M1.

We prepared metabolite M1 from urine samples by semipreparative HPLC and its purity showed more than 98% as analyzed by HPLC (Figure 4). Then we confirmed the structure of M1 by NMR spectroscopy. The ^1^H and ^13^C NMR data for M1 and leonurine were described in Table 3. In ^13^C NMR data, except for the aglycone part of M1 approximately similar to that of leonurine, we found the presence of a glucuronic acid unit [*δ* 104.7 (C-1′), 75.9 (C-2′), 79.2 (C-3′), 74.2 (C-4′), 78.0 (C-5′), and 177.5 (C-6′)] [25] in the structure of metabolite M1. The ^1^H NMR data showed a single proton doublet signal [*δ* 4.99 (H-1′)] which showed a cross signal with C-1′ (*δ* 104.71) on HSQC spectra (Figure 5(a)). Evidence of its coupling constant of the anomeric proton (*J* = 7.44 Hz) showed the presence of a typical **β**-anomeric configuration of the glucuronic acid. The HMBC spectra showed cross peaks between C-10 (*δ* 140.03) and H-1′ (*δ* 4.99) (Figure 5(b)). Thus, M1 was characterized as leonurine-10-O-**β**-D-glucuronide (Figure 6).

## 4. Discussion

A total of three metabolites of leonurine including one phase I metabolite (M3) and two phase II metabolites (M1 and M2) were identified in rat after oral dosing. Generally, glucuronidation is a major drug-metabolizing reaction in humans and accounts for approximately 40–70% of xenobiotic elimination. Many classes of drugs are substrates for this pathway. Biotransformation of drugs generally increases their polarity which can accelerate the excretion of parent drug [26]. In this study, metabolite M1, the O-glucuronidation of parent, is the lead metabolite of leonurine in rat after oral administration. We observed that the level of M1 was far higher (approximately 20-fold) than parent drug in the pooled plasma samples after oral administration at 30 mg/kg. Also, an overwhelming portion of M1 was detected both in bile and urine samples after oral dosing. These results predicted that the bioavailability of leonurine after oral administration may be very low and the oral administered parent drug may undergo first pass metabolism (intestinal or hepatic) to form the metabolite M1. In reversed HPLC system, we observed that the retention time of M1 is shorter than parent drug which indicated that the chemical polarity of M1 is greater than parent drug. Metabolite M1 can be excreted into urine and bile. However, M1 was not recovered in feces; we inferred that M1 in intestinal tract derived from biliary excretion or probably via intestinal metabolism may be absorbed into circulation again and finally eliminated into urine. Because glucuronidation is referred to deactivation and rapid excretion in most cases, a possible drug effect of glucuronide metabolites is frequently neglected. However, this perspective can be wrong, for example, glucuronide metabolite can prolong the pharmacological effect of the parent drug trough an enzymatic or nonenzymatic hydrolysis even if a glucuronide itself has no biological effects. In addition, in some cases glucuronidation was reported to have pharmacological effect (such as morphine-6-glucuronide, digitoxin- and digoxin-glucuronides) and may be associated with toxicity (such as N-O-glucuronides and the acyl glucuronides) [27]. Oral administration of leonurine at dose of 30 mg/kg was reported to have cardioprotective and neuroprotective activity in our laboratory [12, 17]. Since O-glucuronide was the lead metabolite of leonurine after oral dosing, it is very interesting and necessary to figure out what effect of this glucuronide metabolite may be exerted in next study.

Metabolites M2 and M3 were the minor metabolites *in vivo*. Metabolite M2, the sulfate conjugate of leonurine, can be excreted into bile and urine but was not recovered in feces, which suggested that M2 may have enterohepatic circulation phenomenon. Therefore, the mainly elimination route of M2 is urinary excretion. M3, O-demethylated leonurine, was the unique phase I metabolite of leonurine in this study. M3 can be excreted into bile, urine, and feces. M3 recovered in feces was possibly derived from the intestinal metabolism and biliary excretion. The fecal excretion appears to be the major route for the elimination of M3 based on the fact that M3 recovered in feces was far than that in urine.

## 5. Conclusions

In summary, an approach of HPLC/MS/MS was successfully developed and applied to identification of metabolites of leonurine in rat. For the first time a total of three metabolites including two phase II metabolites (M1 and M2) and one phase I metabolite (M3) of leonurine in rat *in vivo* samples were identified. Leonurine was mainly metabolized *in vivo* by glucuronidation, secondarily by sulfation and demethylation. The possible elimination route of leonurine *in vivo* was tentatively elucidated. The structure of the leading metabolite (M1) was further characterized as leonurine-10-O-**β**-glucuronide by NMR analysis. In the future, M1 will have to be synthesized chemically to obtain large quantity of compound for bioactivity study *in vitro *and* in vivo. *In addition, other metabolites such as sulfate conjugate and demethylated metabolite will have to be prepared for conclusive identification by NMR spectroscopy.

## Figures and Tables

**Table 1:** The metabolic profile of leonurine in various rat biological samples.

Biological sample	Mean percentage of metabolites against total counts (%)^a^				Total peak areas (counts)^b^
	M1	M2	M3	M0 (Parent)	
Plasma^c^	94.59	0.70	N.D.	4.71	3.30 × 10^6^
Bile^d^	80.38	1.84	0.28	17.50	5.49 × 10^7^
Urine^e^	77.90	0.21	0.08	21.81	2.79 × 10^7^
Feces^e^	N.D.	N.D.	1.73	98.27	1.70 × 10^7^

N.D.: not detected.

^a^Calculated as (peak area of each compound/total peak areas of all compounds) × 100.

^b^The peak areas of parent and each metabolite were obtained under MRM scan mode.

^c^The pooled plasma samples collected from 6 rats after oral dosing of leonurine.

^d^The 0–8 h bile samples collected from 6 bile duct-cannulated rats after oral dosing.

^e^The 0–16 h samples collected from 6 rats after oral dosing.

**Table 2:** Mass spectra data and proposed structures of leonurine metabolites in rat.

Metabolites	Retention time (min)	Protonated molecule [M + H]^+^	MS^*n*^ product ion	Optimal MRM transitions	Description of metabolites
M0	8.1	312	295, 253, 181, 153, 132, 114, 97	312/181	Parent
M1	4.9	488	312, 295, 253, 181, 132, 114, 97	488/312	Glucuronidation
M2	6.9	392	312, 295, 253, 181, 153, 132, 114, 97	392/312	Sulfation
M3	7.2	298	281, 167, 132, 114, 97	298/167	O-demethylation

**Table 3:** The ^13^C and ^1^H NMR data for M1 and leonurine.

Number	Carbon signals^a,b^		Proton signals^a,b^	
	Leonurine	M1	Leonurine	M1
1	157.3	159.2		
2	40.9	43.2	3.15 (t, 2H, *J* = 6.65 Hz)	3.04 (t, 2H, *J* = 6.65 Hz)
3	26.4	27.1	1.73 (m, 2H)	1.62 (t, 2H, *J* = 7.14 Hz)
4	26.8	27.5	1.58 (m, 2H)	1.52 (quintet, 2H, *J* = 6.96 Hz)
5	63.8	68.1	4.26 (t, 2H, *J* = 6.16 Hz)	4.11 (t, 2H, *J* = 6.06 Hz)
6	166.6	170.1		
7	124.6	128.6		
8 and 12	105.4	109.6	7.22 (s, 2H)	7.05 (s, 2H)
9 and 11	147.8	154.6		
10	138.9	140.0		
13 and 14	56.6	58.7	3.82 (s, 6H)	3.68 (s, 6H)
1′^c^		104.7		4.99 (d, 1H, *J* = 7.44 Hz)
2′		75.9		3.36–3.45 (m, 1H)
3′		79.2		3.48 (t, 1H, *J* = 4.50 Hz)
4′		74.2		3.36–3.45 (m, 1H)
5′		78.0		3.36–3.45 (m, 1H)
6′		177.5		

^a^All spectra were recorded on a Bruker Avance 400 spectrometer, in D_2_O.

^b^The carbon and proton signals were assigned on ^1^H NMR, ^13^C NMR, HMBC, and HSQC.

^c^Carbon number of glucuronic acid moiety.

## Abbreviations

HPLC/MS/MS: High performance liquid chromatography coupled with tandem mass spectrometry
IDA: Information dependent acquisition
XIC: Extracted ion chromatogram
MRM: Multiple reaction monitoring
EPI: Enhanced product ion
NL: Neutral loss
PreI: Precursor ion
CE: Collision energy
DP: Declustering potential
DFT: Dynamic fill time
HMBC: Heteronuclear multiple bond coherence
HSQC: Heteronuclear single quantum coherence.

## References

[1] Hu S (1976). A contribution to our knowledge of Leonurus L., i mu ts’ao, the Chinese motherwort. *The American Journal of Chinese Medicine*.

[2] Sun J, Huang SH, Zhu YC (2005). Anti-oxidative stress effects of *Herba leonuri* on ischemic rat hearts. *Life Sciences*.

[3] Wang ZS, Li DW, Xia WJ, Qiu HQ, Zhu LY (1988). The therapeutic effect of *Herba leonuri* in the treatment of coronary myocardial ischemia. *Journal of Traditional Chinese Medicine*.

[4] Loh KP, Huang SH, Tan BKH, Zhu YZ (2009). Cerebral protection of purified *Herba leonuri* extract on middle cerebral artery occluded rats. *Journal of Ethnopharmacology*.

[5] Xie C-X, Yang Y-Q, Lu J-P, Tang M, Zhou W (2007). Protective effect of Yimucao (*Herba leonuri*) injection against cerebral ischemia: an experimental study in mice and rats. *Nan Fang Yi Ke Da Xue Xue Bao*.

[6] Yeung HW, Kong YC, Lay WP, Cheng KF (1977). The structure and biological effect of leonurine. A uterotonic principle from the Chinese drug, i mu ts’ao. *Planta Medica*.

[7] Chang CF, Li CZ (1986). Experimental studies on the mechanism of anti-platelet aggregation action of motherwort. *Zhong Xi Yi Jie He Za Zhi*.

[8] Chen C-X, Kwan C-Y (2001). Endothelium-independent vasorelaxation by leonurine, a plant alkaloid purified from Chinese motherwort. *Life Sciences*.

[9] Liu XH, Pan LL, Gong QH, Zhu YZ (2010). Antiapoptotic effect of novel compound from *Herba leonuri- Leonurine* (SCM-198): a mechanism through inhibition of mitochondria dysfunction in H9c2 cells. *Current Pharmaceutical Biotechnology*.

[10] Liu X-H, Chen P-F, Pan L-L, Silva RD, Zhu Y-Z (2009). 4-Guanidino-n-butyl syringate (Leonurine, SCM 198) protects H9c2 rat ventricular cells from hypoxia-induced apoptosis. *Journal of Cardiovascular Pharmacology*.

[11] Xin H, Liu XH, Zhu YZ (2009). *Herba leonurine* attenuates doxorubicin-induced apoptosis in H9c2 cardiac muscle cells. *European Journal of Pharmacology*.

[12] Liu X, Pan L, Gong Q, Zhu Y (2010). Leonurine (SCM-198) improves cardiac recovery in rat during chronic infarction. *European Journal of Pharmacology*.

[13] Liu XH, Pan LL, Chen PF, Zhu YZ (2010). Leonurine improves ischemia-induced myocardial injury through antioxidative activity. *Phytomedicine*.

[14] Liu X-H, Xin H, Hou A-J, Zhu Y-Z (2009). Protective effects of leonurine in neonatal rat hypoxic cardiomyocytes and rat infarcted heart. *Clinical and Experimental Pharmacology and Physiology*.

[15] Liu XH, Pan LL, Deng HY (2013). Leonurine (SCM-198) attenuates myocardial fibrotic response via inhibition of NADPH oxidase 4. *Free Radical Biology & Medicine*.

[16] Qi J, Hong ZY, Xin H, Zhu YZ (2010). Neuroprotective effects of leonurine on ischemia/reperfusion-induced mitochondrial dysfunctions in rat cerebral cortex. *Biological and Pharmaceutical Bulletin*.

[17] Loh KP, Qi J, Tan BK, Liu XH, Wei BG, Zhu YZ (2010). Leonurine protects middle cerebral artery occluded rats through antioxidant effect and regulation of mitochondrial function. *Stroke*.

[18] Liu H, Zhang X, Du Y (2012). Leonurine protects brain injury by increased activities of UCP4, SOD, CAT and Bcl-2, decreased levels of MDA and Bax, and ameliorated ultrastructure of mitochondria in experimental stroke. *Brain Research*.

[19] Tolonen A, Turpeinen M, Pelkonen O (2009). Liquid chromatography-mass spectrometry in *in vitro* drug metabolite screening. *Drug Discovery Today*.

[20] Holčapek M, Kolářová L, Nobilis M (2008). High-performance liquid chromatography-tandem mass spectrometry in the identification and determination of phase I and phase II drug metabolites. *Analytical and Bioanalytical Chemistry*.

[21] Liu Z, Zhang Y, Hua YF, Covey JM, Benbrook DM, Chan KK (2008). Metabolism of a sulfur-containing heteroarotionoid antitumor agent, SHetA2, using liquid chromatography/tandem mass spectrometry. *Rapid Communications in Mass Spectrometry*.

[22] Cheng KF, Yip CS, Yeung HW, Kong YC (1979). Leonurine, an improved synthesis. *Experientia*.

[23] Guo J, Gu C, Zhou D, Elmore CS, Bui KH, Grimm SW (2011). *In vitro* and *in vivo* metabolism of a selective *δ*-opioid receptor. *Drug Metabolism and Disposition*.

[24] Liang Y, Wang G, Xie L, Sheng L (2011). Recent development in liquid chromatography/mass spectrometry and emerging technologies for metabolite identification. *Current Drug Metabolism*.

[25] Cui L, Qiu F, Yao X (2005). Isolation and identification of seven glucuronide conjugates of andrographolide in human urine. *Drug Metabolism and Disposition*.

[26] Wells PG, Mackenzie PI, Chowdhury JR (2004). Glucuronidation and the UDP-glucuronosyltransferases in health and disease. *Drug Metabolism and Disposition*.

[27] Shipkova M, Wieland E (2005). Glucuronidation in therapeutic drug monitoring. *Clinica Chimica Acta*.

